# Clinical Requirements of Future Patient Monitoring in the Intensive Care Unit: Qualitative Study

**DOI:** 10.2196/13064

**Published:** 2019-04-30

**Authors:** Akira-Sebastian Poncette, Claudia Spies, Lina Mosch, Monique Schieler, Steffen Weber-Carstens, Henning Krampe, Felix Balzer

**Affiliations:** 1 Department of Anesthesiology and Intensive Care Medicine Charité – Universitätsmedizin Berlin (corporate member of Freie Universität Berlin, Humboldt-Universität zu Berlin, and Berlin Institute of Health) Berlin Germany; 2 Einstein Center Digital Future Berlin Germany

**Keywords:** patient monitoring, digital health, qualitative research, intensive care unit, intensive care medicine, multidisciplinary, user-centered design, design thinking, digital literacy, grounded theory

## Abstract

**Background:**

In the intensive care unit (ICU), continuous patient monitoring is essential to detect critical changes in patients’ health statuses and to guide therapy. The implementation of digital health technologies for patient monitoring may further improve patient safety. However, most monitoring devices today are still based on technologies from the 1970s.

**Objective:**

The aim of this study was to evaluate statements by ICU staff on the current patient monitoring systems and their expectations for future technological developments in order to investigate clinical requirements and barriers to the implementation of future patient monitoring.

**Methods:**

This prospective study was conducted at three intensive care units of a German university hospital. Guideline-based interviews with ICU staff—5 physicians, 6 nurses, and 4 respiratory therapists—were recorded, transcribed, and analyzed using the grounded theory approach.

**Results:**

Evaluating the current monitoring system, ICU staff put high emphasis on usability factors such as intuitiveness and visualization. Trend analysis was rarely used; inadequate alarm management as well as the entanglement of monitoring cables were rated as potential patient safety issues. For a future system, the importance of high usability was again emphasized; wireless, noninvasive, and interoperable monitoring sensors were desired; mobile phones for remote patient monitoring and alarm management optimization were needed; and clinical decision support systems based on artificial intelligence were considered useful. Among perceived barriers to implementation of novel technology were lack of trust, fear of losing clinical skills, fear of increasing workload, and lack of awareness of available digital technologies.

**Conclusions:**

This qualitative study on patient monitoring involves core statements from ICU staff. To promote a rapid and sustainable implementation of digital health solutions in the ICU, all health care stakeholders must focus more on user-derived findings. Results on alarm management or mobile devices may be used to prepare ICU staff to use novel technology, to reduce alarm fatigue, to improve medical device usability, and to advance interoperability standards in intensive care medicine. For digital transformation in health care, increasing the trust and awareness of ICU staff in digital health technology may be an essential prerequisite.

**Trial Registration:**

ClinicalTrials.gov NCT03514173; https://clinicaltrials.gov/ct2/show/NCT03514173 (Archived by WebCite at http://www.webcitation.org/77T1HwOzk)

## Introduction

### Background

In decades to come, demographic developments and an increasing number of comorbidities will lead to an ever-rising number of chronically ill patients in need of intensive care treatment [1]. Moreover, health care institutions are highly challenged with rising workloads, due to a shortage of medical staff and an increasing financial burden [2]. Within this context, rapid and sustainable implementation of advanced digital technologies could mitigate this development.

Continuous monitoring of patients is one of the most essential components in intensive care medicine: first, to notice critical changes of patients’ health statuses, and second, to guide daily intensive care therapy [3]. Its implementation led to significant improvements in patient safety in the intensive care unit (ICU) [4]. Notably, in comparison with other medical devices, patient monitoring is used by a multidisciplinary team of physicians, nurses, and respiratory therapists.

With advances in information and communication technologies (ICTs) and medical device technologies, new options for patient monitoring are being introduced that may potentially improve patient safety [5]. However, most of the monitoring devices used today, such as the electrocardiogram (ECG) or invasive blood pressure measurement, were already available in the 1970s, using alarm thresholds for single sensors [6,7]. Nowadays, technologies to remotely monitor patients are available, such as wireless monitoring sensors (eg, ECG, pulse oximetry [8,9], and hemoglobin [10]), noninvasive measurement of hemodynamic parameters (eg, blood pressure and cardiac output [11]), as well as mobile communication devices (eg, mobile phones and tablets) [12-14]. Furthermore, clinical decision support systems (CDSS) based on artificial intelligence can assist physicians by analyzing multiple parameters to detect early indications of sepsis, respiratory failure, or bleeding [15,16].

Despite these technological developments, the introduction of novel patient monitoring applications in the ICU remains a lagging process compared to other industry sectors [17,18]. The manifold reasons for this could be rooted in a mismatch of expectations and assumptions by clinical users and manufacturers about novel patient monitoring [19,20].

### Aim

This qualitative study evaluated statements by ICU staff—physicians, nurses, and respiratory therapists—on current patient monitoring. This study also evaluated the staff’s expectations for future technological developments to explore clinical requirements and barriers to the implementation of a novel monitoring system. We aimed to explore desires, concerns, and perceived challenges of ICU staff on patient monitoring that may stimulate rapid and sustainable technological adaption in the ICU.

## Methods

### Ethics Approval and Consent to Participate

Ethical approval for this study was provided by the ethics committee of the Charité—Universitätsmedizin Berlin, Germany (EA1/031/18). All participants gave their consent prior to the study.

### Setting

This study was conducted at three ICUs of a German university hospital as a preliminary study of the implementation of the Vital Sync virtual patient monitoring platform 2.4, developed by Medtronic plc. This new system was installed in one of the three ICUs to monitor patients remotely and was utilized after completion of data collection for this study. In all three ICUs, the Philips IntelliVue patient monitoring system was installed at the time of the study (MX800 software version M.00.03; MMS X2 software version H.15.41-M.00.04). The COPRA 5 patient data management system (PDMS), developed by COPRA System GmbH, was used in all ICUs.

### Research Team and Study Design

The research team consisted of a postdoctoral researcher with a background in anesthesiology, geriatrics, intensive care medicine, and digital health (ASP); a senior medical student with a strong affinity for digital health (LM); a professor for digital health, who is a consultant anesthesiologist and a computer scientist (FB); a psychologist (HK); a head nurse (MS); the ICU senior consultant (SWC); and the department’s head of staff (CS). To maintain reflexivity, the research team challenged established assumptions in discussions and shared diaries throughout the study.

We chose an inductive, exploratory, qualitative research approach using semistructured interviews as described elsewhere [21-23]. The inductive approach allowed us to simultaneously collect and analyze data to see if any patterns emerged that would influence the study design.

### Data Collection

Between April and May 2018, ASP and LM conducted face-to-face semistructured interviews with 5 physicians (4 women, 80%), 6 nurses (2 women, 33%), and 4 respiratory therapists (1 woman, 25%) from the ICU. The median of ICU experience was 4 years (range 2-15) for physicians, 6 years (range 1-14) for nurses, and 9 years (range 2-18) for respiratory therapists. Purposive sampling was employed to ensure an evenly distributed variety of professional staff.

The interview design was based on the research question and developed by the research team through consultation of further experts from intensive care medicine and psychology. Pilot interviews did not alter the questions. The developed questions were used as a guide for the interviews, giving the interviewers the freedom to change their weight or phrasing (see Textbox 1). Additionally, the order of the first three questions could be changed. The interviews were conducted during breaks between patient care in the ICU, were recorded and transcribed verbatim by the interviewers, and were reviewed by the researcher who had not done the transcription. Median interview length was 13 minutes (range 8-26).

### Data Analysis

After the completion of five interviews, we began analyzing the data through an inductive approach by means of the grounded theory [24]. Codes that were generated through line-by-line coding of three particularly different interviews resulted in a category system (see Multimedia Appendix 1) that was adjusted and extended by analyzing further interview transcripts (see Multimedia Appendix 2). All coding was performed using the MaxQDA 2018 qualitative data analysis software. The first five interviews were coded twice by two independent researchers (ASP and LM). Inconsistencies between coders were discussed in meetings among the research team until a mutual agreement was achieved. All following transcripts were coded by one researcher and the codes validated by another researcher.

**Figure 1:**
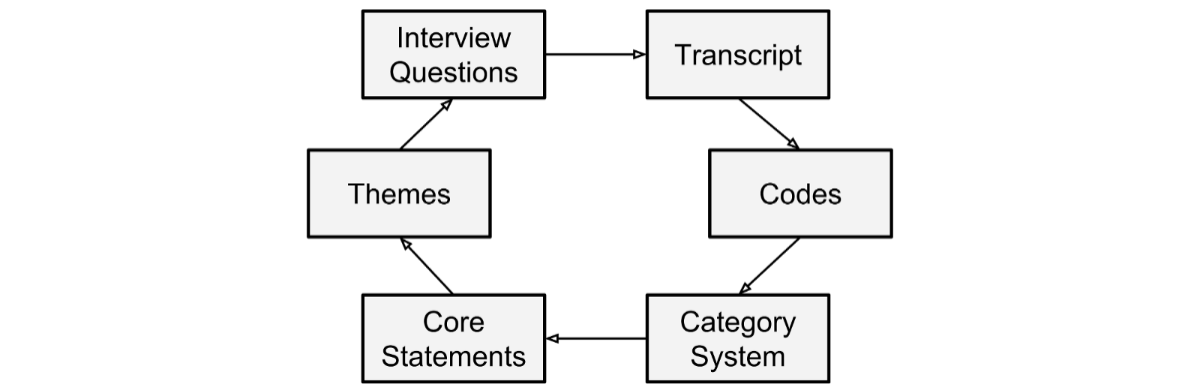
A feedback loop adapted the weight and order of the interview questions through parallel data collection and evaluation as previously described [25].

After completion of coding, the research team reviewed and summarized each core statement to extract themes that were relevant to the study objective. Throughout the process of data analysis, the weight and phrasing of all questions and the order of the first three questions asked during the interviews were adapted using a feedback loop as previously described [25] (see Figure 1). Data collection was finalized when no new codes were identifiable from new interviews [26]. Out of each category, representative statements were selected and translated into English.

The datasets generated and analyzed during this study are not publicly available due to reasons of data privacy; however, they are available from the corresponding author (FB) upon reasonable request.

**Guide for intensive care staff interviews.**

Interview questions:

- How often do you interact with the current patient monitoring system and which features do you use?
- Regarding the current patient monitoring system, is there anything that you find particularly useful? What suggestions for improvement do you have?
- Given endless financial and technical resources, what would your future patient monitoring system look like?
- Would you consider using a tablet for your clinical work regarding remote patient monitoring? In which situations would you use it?
- Would you consider using a clinical decision support system for your clinical routine?
- In your clinical workflow, is it important to have a graphical visualization of patients’ vital parameters and their trends? Do you consider trend graphics of the patient monitoring system useful for shift handovers?
- What is more important to you: usability or number of features?

## Results

### Summary

**Figure 2:**
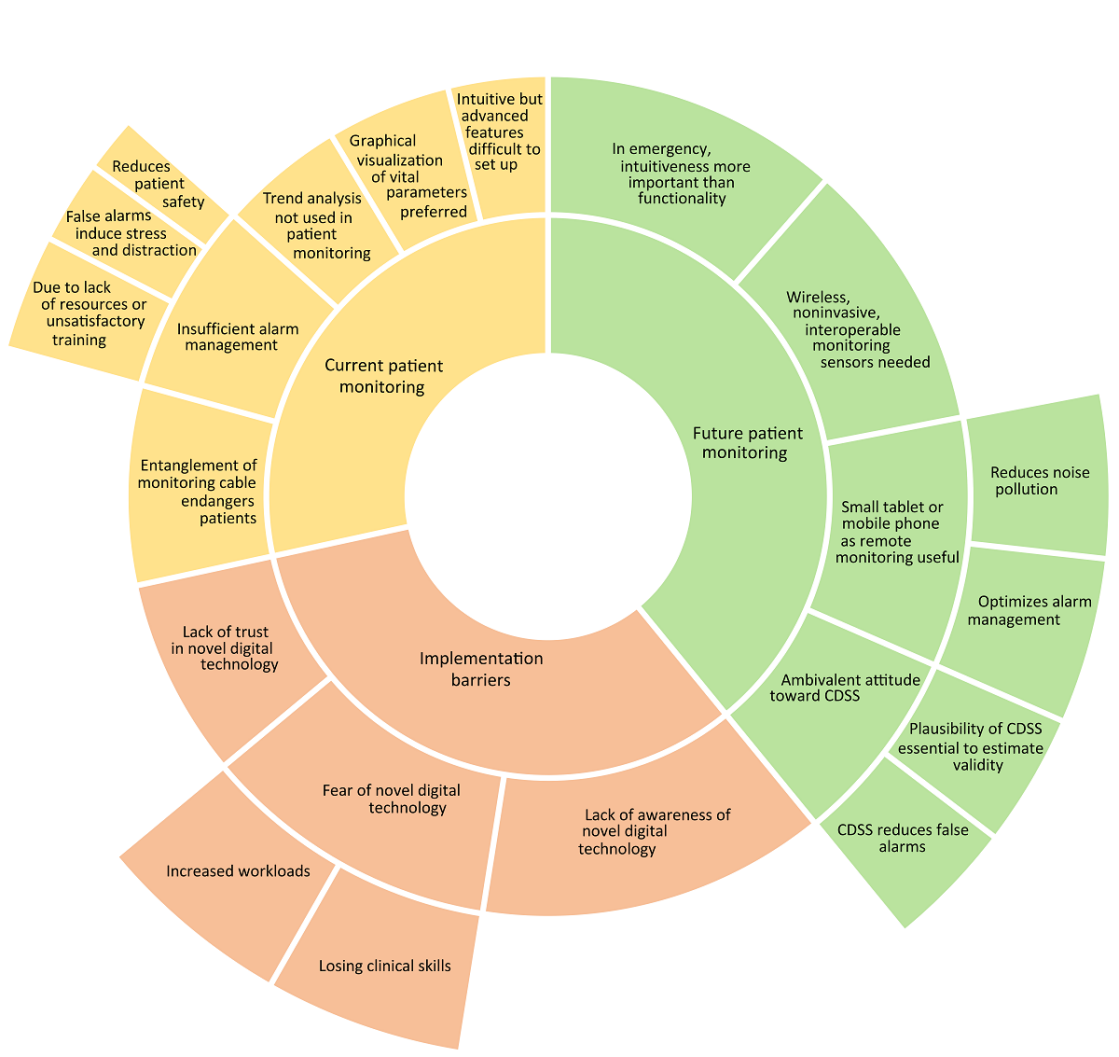
Within three categories (inner ring), 12 themes (middle ring) were identified and specified (outer ring) to reflect the requirements of a novel patient monitoring technology from the view of intensive care staff. CDSS: clinical decision support system.

This qualitative study was constructed based on 15 interviews with ICU staff regarding the complexity of patient monitoring in the ICU. According to our study objectives, resulting codes were classified into three main categories: (1) current patient monitoring, (2) future patient monitoring, and (3) barriers to implementation of novel patient monitoring. In the sunburst diagram (see Figure 2), the 12 most-relevant themes (middle ring) within the three categories (inner ring) are visualized and specified (outer ring).

Most participants saw a need for improvement of patient monitoring in the ICU through novel technology, not only for enhanced efficiency in routine processes, but also to improve patient safety, quality of care, staff satisfaction, and quality of life for patients in the ICU as well as after discharge. Self-evaluation by participants regarding technological savviness using a Likert scale, with scores ranging from 1 (*no affinity for technology*) to 5 (*high affinity for technology*), resulted in the following median scores: physicians, 3.5 (range 2.0-5.0); nurses, 2.8 (range 1.5-4.0); and respiratory therapists, 3.8 (range 3.5-5.0).

### Current Patient Monitoring

The interviewed ICU staff rated the software usability of the current patient monitoring as *good* with special emphasis on intuitiveness and uniformity. Standard features such as display of vital parameters and configuration of alarm thresholds were easy to use, however, advanced settings were considered difficult to set up without training.

It's sometimes very difficult to get all the parameters that I actually want on a monitor...Partly it's very complicated to be able to adjust the monitor quickly and effectively. So I often have the situation that I am called in by the nursing staff because they don't manage to display the parameters on the monitor that I would like to see. And then it costs me 20 minutes of work that is wasted during the day.

Interview 13, physician

For the visualization of single parameters, a graphical curve was stated to be essential for faster clinical interpretations and to ensure the validity of sensor measurements. All professional groups stated that they rarely use trend analysis on the patient monitoring device. Instead, the PDMS was used, as it provides other clinical data along with trends of vital parameters.

Concerning patient monitoring features used by ICU staff, alarm management was mentioned most frequently. Nurses and respiratory therapists would regularly adjust alarm thresholds to current patient conditions. However, alarm fatigue or “cry wolf” situations (ie, multiple alarms going off at the same time) were considered as a major deficit of the current system, leading to stress in patients and staff and, potentially, reduced patient safety. Reasons for this were stated as (1) technical: difficult to distinguish between false and critical alarm, and susceptibility to error of the ECG, peripheral capillary *oxygen* saturation (SpO_2_), and end-tidal carbon dioxide (etCO_2_) sensors; (2) patient related: interference of artefacts related to delirium (ie, movement), sepsis (ie, centralized circulation), or high perspiration; and (3) ICU staff related: inadequate alarm hygiene due to lack of staff training with patient monitoring and lack of staff resources.

Alarm management is rather a big problem in the intensive care unit; some people set the alarm limits very tightly, which often leads to false alarms. I think it's important to work on the alarm management within the team...especially at night, also the sound for the patients. When the patient is supposed to sleep and then the monitor beeps all the time...

Interview p02, nurse

Too little alarm hygiene is being done. This is not due to the laziness of the people, but simply due to the staff situation; there are too few nurses, too few doctors. Therefore, it just beeps very often. And the monitor can't distinguish; is this critical or not? It gets its limits set, and if you've had an alarm five times because the patient is moving, and therefore the heart rate is supposedly elevated, you won't look at it the sixth time, but maybe there is something else. Yes, that's a bit of a problem, because one or the other critical situation is only recognized very late.

Interview 11, respiratory therapist

Long distances and an angled architecture of the ICU along with missing additional patient monitoring displays at strategic positions (eg, corridor and doctor’s office) were indicated to possibly lead to critical situations. Furthermore, all interviewees criticized the entanglement of cables, especially in situations such as bedding and transport, posing a major patient safety issue.

### Future Patient Monitoring

Participants from all professional groups emphasized the importance of intuitiveness and usability of a future patient monitoring system, especially in an emergency, with options to add more advanced and individual settings.

So if you want to use something like that, it would be good to have more functions and individualize it...Because, I think to myself, it is precisely because of the fact that there are so many different professional groups on the move here, that a senior physician in the department may also have completely different things that he finds important than perhaps a respiratory therapist or another specialist.

Interview 12, respiratory therapist

It all has to be self-explanatory in my eyes because we have too many devices that are complicated, so it would be nice if it was very user-friendly.

Interview 7, respiratory therapist

Future conceptions were more accurate in measurements, while at the same time less invasive, wireless, and with better interoperability between medical devices; for example, access to PDMS through patient monitoring.

How do you imagine the monitoring system of the future?

Interview 11, interviewer

Capture more values with less effort. So less invasive and a little more accurate, yes.

Interview 11, respiratory therapist

In any case, a wireless transmission of the monitor would be great. Because this would of course have a clear advantage for the patient in terms of mobility.

Interview 12, respiratory therapist

Participants from all interviewed professional groups believed that using mobile communication technology, such as tablet computers or mobile phones, as remote patient monitoring devices could increase patient safety, reduce the length of stay in the ICU, and improve job satisfaction.

I absolutely believe it [remote patient monitoring] is a step in the right direction. It benefits the patients, after all. And in the best case, it makes the work easier.

Interview p02, nurse

A reduction of stress through remote patient monitoring, in both ICU staff and patients, was pointed out and justified by optimized alarm management (ie, the possibility to cancel false alarms from a mobile device and, thus, less noise pollution).

And if I also had the option of canceling [false] alarms while sitting at the PC without having to run to the central system, I think that would make life easier for me. And above all, it would protect the patient. You do not ignore false alarms, or other alarms, which you interpret as false alarms—which can be life-threatening—and that the patient is perhaps less stressed, if he does not hear these alarms constantly at his own bed...I think I'm also preventing delirium.

Interview 13, physician

To reduce distractions of doctors by false alarms, interviewees also proposed an alarm filtering system by the nursing staff and critical alarm transmission to the doctor’s mobile device.

If you get distracted by other things again and again...I think you accomplish less in the time you have. And, therefore, related to your question, of course it is important that you get alerted, but in the end, I see the nursing staff as a certain filter.

Interview 2, physician

For [external staff and new staff members], I actually don't find that bad at all. That they can just say, “Ok, I press a button and know...when the alarm comes, that goes to the doctor...” And that this makes them more relaxed and they don't have to search for him.

Interview 8, nurse

A point of criticism of remote patient monitoring was the fear of less interprofessional communication and less patient contact when the physician is informed via a mobile device and the alarms are canceled remotely. To achieve better teamwork regarding alarm management, training in interprofessional communication was considered necessary.

I also find that a bit difficult, because then the communication just breaks down a bit. Because I like to go to the doc and say, “Hey, here, I noticed that, should I do something now?”

Interview 8, nurse

Staff expectations regarding the implementation of a CDSS, including artificial intelligence in monitoring, were ambivalent; however, an automatic adjustment of alarm thresholds through trend analysis and the CDSS was suggested. Critical attitudes resulted from lack of trust in the CDSS: the interviewees stressed plausibility to estimate the validity of CDSS recommendations in their clinical work routine.

And if I don't understand the physiology behind it, also in humans, and only stick to these theoretically calculated values there, then I think mistakes will occur...So a basic education in the basic understanding of physiology and also of technology, how these limits and parameters and recommendations arise, should be absolutely there.

Interview 13, physician

In terms of hardware design for remote patient monitoring, several interviewees of all professions agreed that a large tablet was applicable for stationary use because it would provide a better overview. However, most of the interviewed staff said they would prefer using a small device, even their own mobile phone, which would offer greater mobility since the pockets of the scrubs are too small for larger devices.

If I had to carry it [the tablet] with me all the time, then it would have to be the size of a scrubs pocket.

Interview 3, nurse

If it is stationary, then rather large [display] to provide a good overview.

Interview 8, nurse

### Barriers to Implementation of Novel Patient Monitoring

We identified a lack of trust in technology as the greatest barrier to the implementation of novel patient monitoring devices in the ICU.

I think it's important to be at the patient's bedside, look at the patient, and not just rely on some kind of monitoring.

Interview 10, physician

ICU staff feared the implementation of new technology in the ICU that would increase workloads in a setting where time and resources are already scarce.

We have a lot of leasing staff [external staff], and we are a newly assembled team—I think it [new technology] would still be difficult to implement here at the moment.

Interview p02, nurse

They demanded more time for using advanced features and for training in new medical devices.

If I had more time, then I would like to have more functions [in patient monitoring] and we must be trained more intensively for using the new [medical] devices.

Interview p02, nurse

While satisfied with the current system, ICU staff reported that new technology seems very complex and they often did not foresee its benefit. By using new technology, they were afraid to lose their clinical skills and have less direct contact with the patient.

I think that we should use our brain, and that it makes sense to be able to rely on your own senses in case of a power failure, darkness, or whatever.

Interview 10, physician

Well, I think that the more you get taken off [by technology], the more you stop thinking. And then an ECG electrode falls off, and people think the patient is asystolic and start to resuscitate.

Interview 4, nurse

Additionally, lack of awareness and education of ICU staff about current technological developments was identified as a potential barrier to implementation.

## Discussion

### Principal Findings

This qualitative interview study provides valuable insights into the understanding of the complexity of patient monitoring in the ICU. For the ICU staff, the current patient monitoring system was intuitive to use for vital sign monitoring, but other features were difficult to set up due to lack of training and staff shortage. Further, ICU staff rated alarm fatigue and entanglement of cables as major threats to patient safety.

For future developments, a more interoperable, intuitive patient monitoring system was demanded with options to add advanced and individual features depending on the patients’ or users’ needs. Vital parameter measurements and alarms should be more specific, while being noninvasive and less obtrusive (eg, wireless). Interestingly, interviewees recognized mobile phones with a large screen as a potential remote patient monitoring device, which could reduce noise pollution, increase patient safety, and lead to enhanced job satisfaction. Additionally, a CDSS based on artificial intelligence could optimize alarm management if plausible for the ICU staff. For a more rapid introduction of novel patient monitoring solutions in the ICU, participants demanded more training in new medical devices.

As a major barrier to the implementation of novel patient monitoring, lack of both trust and awareness for novel, innovative technology was identified. Interviewees also admitted to being afraid to lose their clinical skills as a result of having less interprofessional communication and less contact with the patient due to novel patient monitoring technology.

### False Alarms Endanger Patient Safety

Whereas alarm management is the main feature of patient monitoring used at the study sites, currently neither regular staff training nor a framework for alarm management is established. In the context where “cry wolf” situations with multiple alarms going off at the same time have become the standard environment in the ICU, this is an alarming insight [27]. Of all auditory alarms, up to 99% have been described to be false alarms that do not change patient treatment [28]. These false alarms are a product of a complex interplay between the patient’s condition, the users’ competence, and the technical features of the patient monitoring system. False alarms desensitize clinical staff to critical alarms (ie, alarm fatigue) and pose a major patient safety issue, leading to alarm-related patient deaths every year [29]. According to our study results, patient safety might also be compromised through the constant noise pollution that induces interruptions, stress, and concentration difficulties among the ICU staff.

Although several strategies have been developed to reduce false alarms in the ICU [12,28-31], implementation into a clinical routine is still lacking. Notably, the reduction of alarms due to alarm management strategies ranges from 24% to 88.5% per ICU, indicating the effectiveness of such strategies, including staff training for any ward that uses patient monitoring devices [32-34].

### Interoperability and Usability of Devices in Intensive Care

Today, most acute care medical devices are not designed to interoperate [18]. Remarkably, our results indicate that requirements for future patient monitoring are steadily increasing to more than just monitoring the vital parameters. ICU staff demand a patient monitoring device to interoperate with other medical devices for detailed comparisons of vital parameters and trend analysis in the context of medication, ventilation, fluid balance, and more, as recently suggested by Flohr et al [35]. This could optimize workflow and reduce redundant documentation in the ICU.

In terms of usability, ICU staff expressed their demand for intuitive and reactive systems for clinical use. Although the implementation of electronic applications in health care dates back more than a decade, usability—referring to the efficient, effective, and safe use of technology—is still not fully optimized for clinical use [36,37]. In the ICU, digital applications should not induce stress. Instead, their use should focus the user for efficient, effective, and safe work. In usability research, various simple and low-cost methods are available that should be applied by anyone working in medical device development [38].

For both interoperability and usability, regular adaptation and application of medical device communication (ie, Institute of Electrical and Electronics Engineers [IEEE] 11073) and technical standards (ie, International Electrotechnical Commission [IEC] 60601) to current developments might minimize use-related hazards and risks to patients and ICU staff [39,40].

### Mobile Phones in Intensive Care Routine

The use of tablet computers with access to electronic medical records or multiparameter monitoring has been perceived as beneficial in inpatient settings [35,41]. However, for ICU staff, large tablets were too bulky to carry around due to the small pockets of their scrubs; they instead preferred small tablets that are portable [42] or larger mobile phones for remote patient monitoring in the ICU. This finding may influence further device developments for the ICU and the operating room where scrubs are worn. Recently released foldable mobile phones could be an approach to combine the advantages of pocket-size and large-screen devices [43]. As industry stakeholders are already developing apps for mobile devices in the ICU, more interdisciplinary studies are necessary to obtain early feedback from clinicians, developers, and engineers [12,14].

In the move toward a widespread implementation of telemedicine and remote patient monitoring technology into various health care sectors including the ICU, the mobile phone or tablet computer could easily be deployed for these tasks. ICU staff claimed that the length of stay in the ICU could be reduced through the utilization of remote patient monitoring, which is in line with several recent studies on telemedicine [44,45].

### Clinical Decision Support Systems for Alarm Management

Integration of novel medical devices and technological advances result in a steadily growing amount of data that are being analyzed by ICU staff daily, thus making automated systems based on artificial intelligence a necessity for the future. Although various research projects are focusing on CDSS in the ICU, translation into the clinical routine is lagging far behind [15,46-49].

In our study, participating staff stated that they would utilize a CDSS only if it was plausible and underlying algorithms were readily understandable. A physician also indicated that appropriate training for the application would be useful to avoid misuse. Taking into account that most CDSS are based on complex machine learning methods, explaining the underlying mechanism to intensivists might be challenging. However, participants expressed the necessity to optimize detection of false alarms with a CDSS. Thus, a self-learning alarm system via machine learning might be practicable for the near future [50].

Furthermore, according to interviewees, trend-based alarms might be a useful complement to the traditional threshold-based alarms; this is consistent with a publication by Charbonnier et al, who was able to reduce 33% of false alarms by using a trend-based alarm system in the ICU [51].

### Building Trust in Information and Communication Technology

The most disruptive implementation of ICT in intensive care medicine in the recent past has been the introduction of tele-ICUs, which has been accompanied by several staff acceptance studies [21,52,53]. With the implementation of tele-ICU technology in existing ICUs, ICU staff are not only confronted with novel ICTs, but also with changes in clinical processes, such as teamwork, communication, and staff structure. This is due to the fact that therapy decisions are influenced by external experts, who might be unfamiliar to the ICU staff on site. In this constellation, trust has to be formed first toward the new ICT and in a second step toward the external experts [21]. With respect to our study, similar concerns were reported: after trust in ICTs are established, ICU staff must also get familiar with the CDSS, in contrast to the external (ie, human) experts. Notably, our results did not show any influence of prior experience with technology on the formation of trust [54].

We conclude that ICU staff are ready and willing to use more-advanced ICT devices in intensive care routine. Nevertheless, without adequate and regular training in novel technical and digital devices, even in alarm management, the full potential of digitization will not have been exhausted.

### Digital Literacy

As suggested in recent publications, governments, health care institutions, and universities should include digital health care in the curriculum of high schools, as well as in medical and nursing schools, to ensure that future health care professionals acquire digital literacy [55,56]. Our finding of low tech-savviness among ICU staff indicates that regular staff training with novel medical devices, software, and mobile phone apps may be beneficial for successful implementation of future patient monitoring devices [20,57,58].

Innovation in health care derives from interdisciplinary teamwork with developers and medical engineers [59]. University hospitals, especially, should empower ICU staff to pursue academic research in the context of ICT implementation in the ICU.

### Design Thinking in Health Care

In the context of digitization in health care, novel digital systems often fail after implementation as a result of a lack of user involvement [59]. The importance of validation of novel digital health solutions through early and continuous user involvement is often underestimated by the industry, hospitals, and governments [55]. Reasons for this include lack of financial resources, delays in time to market, or ignorance about how to validate a digital health product [59]. One way to mitigate this issue might be the design-thinking framework as a systematic process that prioritizes empathy for the users with the aim to develop a more comprehensive and effective solution [60]. In situations where the users cannot point out their needs, analyzing their behaviors through a more user-centered qualitative method such as design thinking can provide invaluable insights about their unmet desires [60].

### Limitations

Through the use of a qualitative interview study design, we could identify several novel findings on the themes of patient monitoring from the perspective of ICU staff. However, as a descriptive approach, quantification of statements is not possible by design. When interpreting the results, it is crucial to take into account the small number of participants of a single hospital (ie, three ICUs) and possible biases due to the selection of participants. This makes the generalization to other hospital settings or countries difficult. A follow-up, quantitative, survey-based study with a larger cohort may be conducted on the basis of this study to further consolidate the results.

Moreover, it is not possible to draw conclusions about whether a novel patient monitoring system can improve patients’ quality of life or quality of care in the ICU. Interdisciplinary investigations with patients, their relatives, health care providers, and technicians (ie, IT and engineering) might shed light on this question. Finally, a bias due to the implementation of the Vital Sync virtual patient monitoring platform cannot be excluded with certainty.

### Conclusions

This qualitative study involves core statements by ICU staff in the analysis of current and novel patient monitoring applications in the ICU. In order to introduce more sustainable digital health solutions in the ICU, health care stakeholders might have to focus more on user-derived findings than top-down speculations. By valuing the opinions of health care providers, we may gain their trust to implement novel systems.

In particular, the results on alarm management and mobile devices in the ICU may be used (1) by health care organizations to prepare ICU staff for digital transformation, (2) by research institutes to reduce alarm fatigue, (3) by industry players to embrace medical device usability, and (4) by political stakeholders and decision makers to advance interoperability standards in intensive care medicine.

Our findings should motivate other researchers to conduct qualitative patient- and user-centered research in health care, especially before developing or implementing premature technological solutions.

## Appendix

Multimedia Appendix 1 Category system that was constructed through line-by-line coding of the interview transcripts.

Multimedia Appendix 2 Catalog with quotes from intensive care unit (ICU) staff regarding patient monitoring.

## Abbreviations

BioCog: Biomarker Development for Postoperative Cognitive Impairment in the Elderly
CDSS: clinical decision support system
DFG: Deutsche Forschungsgemeinschaft
DLR: Deutsches Zentrum für Luft- und Raumfahrt eV
ECG: electrocardiogram
etCO_2_: end-tidal carbon dioxide
ICT: information and communication technology
ICU: intensive care unit
IEC: International Electrotechnical Commission
IEEE: Institute of Electrical and Electronics Engineers
PDMS: patient data management system
SpO_2_: peripheral capillary oxygen saturation
