# Supplementary material for: Clinical Requirements of Future Patient Monitoring in the Intensive Care Unit: Qualitative Study
Source: JMIR Med Inform. 2019 Apr 30;7(2):e13064. doi: 10.2196/13064 (PMC6658223; doi:10.2196/13064)
Supplement: Multimedia Appendix 1 [file medinform_v7i2e13064_app1.pdf]

Appendix 1: table of codes

**Demographic Data**
**Sex**
female
male
**IT affinity**
nursing staff
respiratory therapist
physician
**Work experience on ICU**
nursing staff
respiratory therapist
physician
**Perceived Barriers to Implementation**
**No time for training**
**Digital infrastructure lacks functionality**
**Many external nurses - much time for training with new system**
**Communication problems**
**Clinical decision support systems (CDSS)**
difficult to understand technology
no clear benefit perceived
technology not reliable
fear to lose clinical skills
satisfied with existing solution
**Remote patient monitoring (RPM)**
clinical skills better than monitoring
trust in colleagues more than RPM
less direct contact with patients
not thinking about problem solution
skepticism
increased workload
already satisfied with available system
no clear benefit perceived
less distance, more stress
no trust in new monitoring solutions
**Current patient monitoring (PM)**
**Environmental Factors**

- architecture of ICU
  - long distances
- **Usability PM**
  - **Task-specific**
    - ***Central Monitor***
      - usability
        - task-specific
        - technical
          - more monitors needed
    - ***Monitoring other patients from bedside PM***
      - useful
      - patient identification difficult
    - ***Trendanalysis***
        - *Purpose*
          - special questions
          - overview
        - *Usage*
          - daily
          - seldom
            - Only additionally to Patient Data Management System (PDMS)
          - no use
            - using trends in PDMS
                - better visualization
                - more contact to PDMS than PM
    - ***Resp. Monitoring***
      - respiratory weaning
      - spO2 is not sufficient for respiratory monitoring
      - usage of PM and respiratory device
    - ***Alarm Management***
      - *Responsibilities*
      - *Problems*
        - mental issues
          - alarm fatigue
          - stress
        - communication problems
        - too many alarms
          - reasons
            - technical reasons

                    - patient-related
                    - poor alarm hygiene
                        - lack of training (false alarm limits)
                        - lack of resources
        - *Alarm thresholds*
            - check ECG alarm retrospective
            - active change of thresholds
                - frequency
                    - regularly
                        - once per shift
                    - seldom
                - reason
                    - user-oriented
                    - patient or situation oriented
                        - change of therapy
    - ***Routine PM***
        - daily usage
- **Technical**
    - ***Measurement inaccuracies***
        - ECG measurement error
        - spO2 not possible for shock patients
        - artifacts in the respiratory curve
        - CO2 measurement error-prone
    - ***Auditive alarms***
        - alarm prioritization (importance)
    - ***Hardware***
        - entanglement of cables negative
        - respiratory rate through ECG not possible
        - touchscreen
            - touchscreen useful
        - remote controller
            - useful
            - battery empty
        - monitor
            - monitor reflects
    - ***Visualization***
        - *Other medical hardware for visualization*
            - respirator

- *Design*
  - good
    - used to this design
    - color
    - fond
    - uniform
    - good overview
- *Graphic visualization*
  - optimum scale problem
  - number alone is sufficient
  - graphic vs numeric visualization
    - graph important for clinical assessment
    - validity of measurement
    - respiratory rate curve for respiratory assessment
      - loops for respiratory assessment
    - faster and better visualization of trends

Compatibility with other hardware

- small monitor for transports
- PDMS
- ventilator

System settings

- *Individual setting options*
  - visualization
    - display: parameter
    - curve display / amplitude
- *Good basic settings*
- *Advanced functions difficult to set*
  - individual adaptation to the specialist field not well
  - settings take too much time
- *Parameters (see also Future: concrete wishes)*
  - frequently used parameters
  - important parameters are missing (subject-specific)

Intuitiveness and simplicity

Future PM

Social Factors

- education of staff
- enhance team communication
- more helpers for nurses

- **Usability PMS**
  - **Usability and intuitiveness**
    - modular system with complex functions more hidden
    - more functionality for more training time
    - usability more important
      - usability important for emergency
    - usability requirements individually different
    - individually different number of functions
    - less training, more functions
  - **Technical**
    - better transmission of data, interoperability of devices
    - alarm management
      - different alarm tones for different parameters
    - wireless
      - wireless useful
        - for transports
        - enhance mobility of patients
        - comfort for patient
        - less artifacts
        - wireless for patient bedding
    - visualization
      - relevant parameters get bigger in size
      - all parameters in one monitor
      - visualisation at bedside important
    - non-invasive
    - required parameters for general monitoring
      - etCO2 validated
      - modular system with all available parameters
      - BIS
      - non-invasive cardiac index
      - depth of breath
      - no need for more parameters
      - etCO2 for non-intubated patients
  - **Task-specific**
    - ***Remote Control***
      - less patient contact
      - respiratory device
      - remote control of PM

    - useful when alarm cry
    - less workload
- ***Remote PM***
  - usability remote PM
    - *Task-specific*
      - patient-oriented
        - for noise reduction at bed-side
        - increase of patient safety
        - monitoring non-ICU patients for fast track
        - weaning
        - critically ill
        - known patients
      - staff and resources
        - wireless PM for ward rounds
        - for less alarm fatigue
          - forwarding alarm to doctor
          - direct information about alarm reason
          - filtering alarms through nurse
        - when preparing the medication
        - responsible for more than one ward
        - remote PM from home
        - remote PM from on-call room
        - transports
          - for intervention with radioactive substances, radiation
          - CT Transport
        - neighboring patient room
        - rural areas
        - staff shortage
      - for education
      - tablets for handing over
        - will use
        - not needed
    - *Technical*
      - Alarm management RPM
        - voice alarm
        - vibration alarm
        - audio alarm
          - only for vital alarms

				visual alarm		
			video monitoring			
			visualization			
				graphical		
				design		
					color codes for alarms	
			RPM and PDMS in one system			
			parameter for remote PM			
			hardware			
				projection through beamer		
				size of hardware		
					small sized tablet	
						to have free hands
						easy to transport
						remote PM on body
					middle sized tablet	
					big sized tablet	
						if stationary
						better overview
					smartphone better	
				tablets / smartphone in ICU		
					tablets in danger of theft	
					useful	
					not useful	
						no space to carry additional devices
Clinical decision support systems (CDSS)						
	Usability					
		task-specific				
			alarm automatic threshold query			
			sepsis risk assessment			
			CDSS only for doctors			
			automatic trend analysis			
			scores			
			intelligent alarm management			
				CDSS learns from false alarms		
				CDSS automatically adjusts alarm limits		
				CDSS suggests solutions to solve alarm		
				alarm prioritization		

alarm filter
less interdisciplinary communication
pattern recognition of alarms
individualization of alarms
report of alarms
closed loop system in ICU
