# Supplementary material for: Clinical Requirements of Future Patient Monitoring in the Intensive Care Unit: Qualitative Study
Source: JMIR Med Inform. 2019 Apr 30;7(2):e13064. doi: 10.2196/13064 (PMC6658223; doi:10.2196/13064)
Supplement: Multimedia Appendix 2 [file medinform_v7i2e13064_app2.pdf]

Appendix 2: Citations

Table 1: Catalog with citations regarding current patient monitoring

Quote	Summary
"What do you like about the patient monitoring system?" - "That it's intuitive. The monitors are the same everywhere. And that you can still make certain individual settings somehow." - (interview 09 - physician)<br><br>*"What do you like about the current patient monitoring system?" - "The simplicity. That I […] can also figure out [...] myself, how to adjust the values, or how to take vital parameters in or out."* - (interview 08 - nurse)	Intuitiveness and uniformity
"I wouldn't know in a minute how to configure that out [trend analysis]. *Or I would have to just really search for it. Last week I was looking for a Pacer, how to set a Pacer."* - (interview 04 - nurse)<br><br>*"I've been here for about six months. Just now I'm slowly starting to discover the small fine tunings* [of patient monitoring settings] *as well. But I'm still* [...] *having a hard time with it, for example, when we come back from a patient transport and you put this module back in and then the curves are suddenly different than when we left. Then I really have a lot of trouble fixing it the way it should be."* - (interview 05 - nurse)<br><br>*"It's sometimes very difficult to get all the parameters that I actually want on a monitor.* [...] *Partly it's very complicated to be able to adjust the monitor quickly and effectively. So I often have the situation that I am called in by the nursing staff because they don't manage to display the parameters on the monitor that I would like to see. And then it costs me 20 minutes of work that is wasted during the day."* - (interview 13 - physician)	Complicated set up of advanced features
"So here [with the pulse oximetry curve], *such an analysis of individual curves is already important. Because you know that if the pulse oximetry curve does not show a pulse, the value is incorrect sometimes.* [...] *With* $etCO_2$*, I also think it's important to see how the curve looks like, you can often see whether there are problems with ventilation or not, or whether you would have to change the settings at this point. "* - (interview 02 - physician)<br><br>*"I would like to see the curve, too, because especially with the oxygen saturation value, errors are noticed every now and then, not only because of restlessness* [of the patient]*, but also because of bad blood circulation.* [...] *and there, I would like to see; is this real or not? And for that I would just have to see, well, how is the curve looking now, is it blurred, is it much too flat, is it somehow not synchronous.* […] *This means that the curves are quite important for me."* - (interview 06 - nurse)<br><br>*"Just in my field as a respiratory therapist, curves also help me to see certain things better because the values alone indicate nothing."* - (interview 11 - respiratory therapist)<br><br>*"Is the graphical visualization of such curves important to you? For example the* SpO_2 *curve or the blood pressure curve?" – "Yes, it is important to me. Because you get a quick overview."* - (interview 13 - physician)	Graphical visualization important for the clinical work
"Because, of course, I have more contact with COPRA [patient data management system], [...] *I sit at the computer and look at* [...] *the various patient balances and also the corresponding trends.* [...] *For me, this overview is actually sufficient.* [...] *At the moment I wouldn't be able to comprehend how I should gain in knowledge by this - if I do it on the Philips monitor, if I look at the trends there* [...] *that wouldn't give me any insight now, because I actually do have all the data in COPRA. "* - (Interview 02 - Physician)<br><br>*"For me* [that] *is not so relevant in daily practice, so COPRA actually records something like that.* [...] *Well, I know there is the possibility to do trend*	Analysis of vital parameter trend is rather done with the patient data management system

analyses via the Philips monitor, but I personally don't actually use them [...]" - (Interview p02 – nurse)	
"They [alarm thresholds] *have to be checked at the beginning of the shift."* - (interview 04 - nurse)<br><br>*"It is hard to put it into words how often per shift or service - ten, twenty times - it depends - I set my alarm limits, my parameters that I want to see."* - (interview p02 - nurse)	Regular adjustment of alarm thresholds by nurses
"One is distracted by a lot of false alarms and possibly no longer registers a real critical alarm as such, because the alarm was tinkling the whole morning and then one no longer listens. But this is of course the point where it's always difficult to keep the right balance. - (interview p01 - physician)<br><br>*"Alarm management is rather a big problem in the intensive care unit, some people set the alarm limits very tight, which often leads to false alarms. I think it's important to work on the alarm management within the team.* [...] *especially at night also the sound for the patients. When the patient is supposed to sleep and then the monitor beeps all the time..."* - (interview p02 - nurse)<br><br>*"Too little alarm hygiene is being done. This is not due to the laziness of the people, but simply due to the staff situation, there are too few nurses, too few doctors. Therefore, it just beeps very often. And the monitor can't distinguish; is this critical or not. It gets its limits set, and if you've had an alarm five times because the patient is moving, and therefore the heart rate is supposedly elevated, you won't look at it* [when the alarms goes off] *the sixth time, but maybe there is something else. Yes, that's a bit of a problem, because one or the other critical situation is only recognized very late."* - (interview 11 - respiratory therapist)<br><br>*"Sure, there are always false alarms from time to time, yes; invalid measurements because our ECG was not properly sticked or somehow a saturation measurement does not function as it should."* - (interview 12 - respiratory therapist)<br><br>*"This* [cry wolf situation] *is also* [...] *a little bit due to the patient, with restless patients it is simply quite normal that he sometimes plucks off something or moves and then* [the sensors*] just go off, as well as with strongly sweating patients."* - (interview 06 -nurse)<br><br>*"So we have such a mixed patient group here, which is partly also awake and delirious, which also simply removes the things* [the sensors*] and thereby provokes alarms, which do not represent an acute emergency."* - (interview 13 - physician)<br><br>*"As a respiratory therapist, I try to set the alarms in consultation with the nursing staff. On the ventilator certainly, but also on the Philips monitor I often see that important alarms are badly set or forgotten or even deactivated."* - (interview 12 - respiratory therapist)<br><br>*"Besides, of course, one pays too little attention to the threshold settings. But maybe it should get more attention."* - (interview 13 - physician)	Problems with alarm management:<br>• alarm fatigue<br>• cry wolf situations<br><br>result: stress in patients and staff, reduced patient safety
"Depending on how the ward is structured - and in our case rather unfavorable - one does not always have an overview of all patients. In this respect, however, at least one central monitoring system should be visible [all the time, from anywhere on ICU]." - (interview 07 – respiratory therapist)<br><br>*"I think we have a pretty twisty ward here and central patient monitors in the hallway would be very helpful*." - (interview 02 – nurse)<br><br>*"Well, the way from room one to room nine is so long that you don't notice it.* [...] *It would probably make sense to set up such a monitoring system in the*	Angled ward architecture and long distances on ICU make more central monitors necessary

hallway [...] *in the corner, for example, so that you would* [...] *simply have a better overview."* - (interview 01 - physician)	
"That's quite a tangle of cables, which is still very susceptible to artifacts. […] *It is still the case that I have to verify again and again, whether it is a real alarm."* - (interview p01 - physician)<br><br>*"For CT-rides, surgery rides, it's just annoying, all this cable clutter."* - (interview p02 - nurse)<br><br>*"*[I wish to] *have a little less cable when you do any positioning, so not just abdominal positioning, even if you do some other crazy things."* - (interview 11 - respiratory therapist)	Entanglement of cables hinders smooth workflow

Table 2: Catalog with citations regarding expectations of a future patient monitoring

Quote	Summary
"I would personalize it [patient monitoring]. *If you have had a training, you get a password, and then access to the advanced functions. Or the doctor has the extended functions and can adjust them."* - (interview 08 - nurse)<br><br>*"So if you want to use something like that, it would be good to have more functions and individualize it* […]. *Because, I think to myself, it is precisely because of the fact that there are so many different professional groups on the move here, that a senior physician in the department may also have completely different things that he finds important than perhaps a respiratory therapist or another specialist."* - (interview 12 - respiratory therapist)	Individual settings and advanced features of patient monitoring
"It all has to be self-explanatory in my eyes because we have too many devices that are complicated, so it would be nice if it was very user-friendly." - (interview 07 - respiratory therapist)	Usability important for medical device
"How do you imagine the monitoring system of the future?" – "Capture more values with less effort. So less invasive and a little more accurate, yes." - (interview 11 - respiratory therapist)	Future patient monitoring is less invasive and more functional
"So the best thing would be really if you could have the patient in the bed without cables." - (interview 13 - physician)<br><br>*"What do I wish for? Well, actually I'm already satisfied if it works wireless, yes."* - (interview 03 - nurse)<br><br>*"In any case, a wireless transmission of the monitor would be great. Because this would of course have a clear advantage for the patient in terms of mobility."* - (interview 12 - respiratory therapist)	Wish for wireless monitoring
"And that this data - I mean it is already stored now, so if you reinstall the module, you can recognize old trends, but for example [the values from a] *CT transport* [are] *not transferred retrospectively. It is not entered into the COPRA* [patient data management system] *file. Yes, something like that would certainly be quite good, that one can document something like this without gaps, because it is stored in the module."* - (interview 02 - physician)<br><br>*"With the infusion pumps, for example, there could be a connection to COPRA, our documentation system, so that as soon as I change something on the infusion pump, or with COPRA, it adjusts automatically. [...] How often do you change catecholamines, but don't enter it right away? That would be a great link that would also make your work easier, I could imagine."* - (interview p02 - nurse)	Need for better interoperability of devices

"I think it would save many patients from lying in the intensive care unit if you could say: ok this is an OSAS [obstructive sleep apnea] *patient who cannot sleep here anyway because it's so noisy here, but I have to monitor him. Then it probably makes more sense to telemetrically monitor him, in order to be able to intervene in case of an emergency."* - (interview p01 - physician)<br><br>*"Actually, I think it* [remote patient monitoring] *is good* [...] *because I previously worked in a house where I did a lot of on-call duty and I also had to go a lot to other wards, for example to do an anesthesia talk, and the intensive care unit then was without doctor supervision. And that's exactly the point where I would have liked to have my tablet with me to see if everything was okay. Or when I get an alarm - or a phone call through the nurse who asks me for help, I can already say - ok, now do that and that. Because I can tell from my tablet what the problem is."* - (interview 13 - physician)<br><br>*"I absolutely believe it* [remote patient monitoring] *is a step in the right direction. It benefits the patients, after all. And in the best case it makes the work easier."* - (interview p02 - nurse)<br><br>*"In areas such as Mecklenburg-Vorpommern or in rural areas where help is not there so quickly, it* [remote patient monitoring] *makes sense, I think, to be able to react quickly, especially if you actually know the patient. I believe that you can save lives there."* - (interview 11 - respiratory therapist)	Remote patient monitoring increases patient safety, job satisfaction, reduces length of stay on ICU
"For [external staff and new staff members], *I actually don't find that bad at all. That they can just say ok, I press a button and know* [...] *when the alarm comes, that goes to the doctor* [...]*. And that this makes them more relaxed and they don't have to search for him."* - (interview 08 - nurse)<br><br>*"If you get distracted by other things again and again,* [...] *I think you accomplish less in the time you have. And therefore related to your question: Of course it is important that you get alerted, but in the end I see the nursing staff as a certain filter."* - (interview 02 - physician)	Alarm forwarding through nurses to improve alarm management
"I also find that a bit difficult, because then the communication just breaks down a bit. Because I like to go to the doc and say hey, here, I noticed that, should I do something now?" - (interview 08 - nurse)	Alarm forwarding could lead to less inter-professional communication
"And if I also had the option of canceling [false] *alarms while sitting at the PC without having to run to the central system, I think that would make life easier for me. And above all, it would protect the patient. You do not ignore false alarms, or other alarms, which you interpret as false alarms - which can be life-threatening - and that the patient is perhaps less stressed, if he does not hear these alarms constantly at his own bed. If I get a central alarm at my central monitor and don't always have it at the patient's bedside, then I think I'm also preventing delirium."* - (interview 13 - physician)<br><br>*"Then, on the other hand, the ringing alarm would become a personal alarm, i.e. a friendly voice would say; "look here, that's wrong here" - and then perhaps it would even suggest solutions;* [...] *so that the infusion pumps in the intensive care unit would also be adjusted. If the* [patient] *fluctuates a little within the pressure limits there, that is adjusted, like the insulin pump. That would be advantageous, for example, on the one hand, and on the other hand, that I would not always be so compelled to go in for every small alarm, but that you* [...] *get such an overall report* [...]*; here, that happened, and then you would have to work on it."* - (interview 08 - nurse)<br><br>*"Do you think a system that learns would make sense? So that the monitor says: you have now turned off the alarm for increased frequency five times, shouldn't we set the alarm limit? Such a question that appears after the fifth time?" - "Yes, I wouldn't think it was bad at all. That would be something we could or should try. So somehow you have to get the system and the people to handle these alarms differently."* - (interview 11 - respiratory therapist)	Optimized (intelligent) alarm management leads to a reduction of stress for both patients and healthcare professionals

“To be honest, I find this [application of CDSS] *very difficult because there is a danger that you will only know the numerical limits without the way they were created, and thus will not carry out error analyses. You won’t recognize them. And perhaps also undertake actions that are simply based on false results.”* - (interview 13 - physician)	lack of trust in clinical decision support systems (CDSS)
“And if I don't understand the physiology behind it, also in humans, and only stick to these theoretically calculated values there, then I think mistakes will occur. […] *So a basic education in the basic understanding of physiology and also of technology, how these limits and parameters and recommendations arise, should be absolutely there.”* - (interview 13 - physician)<br><br>*“Is it* [the CDSS] *a black box for you, are you a bit afraid that the machine might calculate something wrong* [...]*?” - “No,* [...] *if I have a control that the parameters arrive correctly, I would have to see them, and then I know whether it calculates them correctly or not.”* - (interview 03 - nurse)<br><br>*“So you want to understand exactly what's being calculated?* [CDSS algorithms]*” - “Exactly. I'm someone who wants to understand things and I'm very happy that technology does a lot for us, but quite often particularly young people rely on technology, on monitoring, instead of looking at the patient. And sometimes, with a bit of experience, you see a problem rather than the person taking care of the patient.”* - (interview 11 - respiratory therapist)	Importance of comprehension of CDSS
“So I think this takes training - so that the colleagues either discuss with the doctor if necessary, or work out together how they adjust the values. The [patient with] *hypertension should not have blood pressure* [alarm] *limits when he tends to surpass them the whole time. This just causes unrest, you should adjust it individually!”* - (interview 08 - nurse)<br><br>*“Well, these are already sensitively set alarm limits which on the one hand make sense, especially for unstable patients, but you just have to somehow train the nursing staff accordingly.”* - (interview 02 - physician)<br><br>"[Regarding alarm fatigue] *I think it's more important to work on our alarm management within the team."* - (interview p02 - nurse)	Wish for increased training of staff with technologies, in communication and teamwork
"Otherwise of course the overview is somehow important, therefore I find a bigger one always better." - (interview p01 - physician)<br><br>*"If it is stationary, then rather large* [display] *to provide a good overview."* - (interview 08 - nurse)<br><br>*"Small tablets are very convenient; you have so much stuff to carry around with you anyway."* - (interview 13 - physician)<br><br>*“If I had to carry it* [the tablet] *with me all the time, then it would have to be the size of a scrubs pocket."* - (interview 03 - nurse)	Big device: better overview, applicable for stationary use<br><br>Small device: greater mobility.
"And, as I said, that's also great, of course, if you have both things together. If you have the tablet, [...] *you also have access to the PDMS data"* - (interview 12 - respiratory therapist)	Patient data management system (PDMS) and monitoring should be remotely accessible on one screen

Table 3: Catalog with the most important citations regarding barriers to implementation of novel patient monitoring.

Quotes	Summary

"I think it's important to be at the patient's bedside, look at the patient and not just rely on some kind of monitoring." - (interview 10 - physician)<br><br>*"Quite often young people rely on monitoring instead of looking at the patient. And with some experience, you see a problem that patient monitoring cannot see"* - (interview 11 - respiratory therapist)<br><br>*"There are a lot of things that are much better observable and assessable for me on patients than just the values that I see in patient monitoring."* - (interview 12 - respiratory therapist)	Lack of trust in new technology
"Well, I think the system is pretty good already." - (interview 04 - nurse)<br><br>*"The visualization is actually ok so far. I don't see how it could be changed"* - (interview 08 - nurse)<br><br>*"Honestly, I think... I think what we have is enough.* [patient monitoring]*"* - (interview 10 - physician)	Satisfied with current situation
"Because I always see, the more complex such calculations are - these are not measured values, but calculations from measured values - the more [data] *is included, the higher the error rate."* - (interview 12 - respiratory therapist)	Fear of too complex system
"If I had more time, then I would like to have more functions [in patient monitoring] *and we must be trained more intensively for using the new* [medical] *devices"* - (interview p02 - nurse)<br><br>*"We have a lot of leasing staff* [external staff], *and we are a newly assembled team - I think it* [new technology] *would still be difficult to implement here at the moment."* - (interview p02 - nurse)	Lack of training / resources
"On the other hand, I don't yet know in which area I would use it [CDSS]. *Where can I detect something out of it that I can't retrieve from the computer or surveillance monitor?"* - (interview 08 - nurse)<br><br>*"I don't miss it* [remote patient monitoring]. *And to be honest, I don't want to have to carry anything around with me."* - (interview 10 - physician)	No perceived benefit
"To be honest, I find this [application of CDSS] *very difficult because there is a danger that you will only know the numerical limits without the way they were created, and thus will not carry out error analyses. You won't recognize them. And perhaps also undertake actions that are simply based on false results."* - (interview 13 - physician)<br><br>*"I think that we should use our brain, and that it makes sense to be able to rely on your own senses in case of a power failure, darkness or whatever."* - (interview 10 - physician)<br><br>*"Well, I think that the more you get taken off* [by technology], *the more you stop thinking. And then an ECG electrode falls off, and people think the patient is asystolic and start to resuscitate."* - (interview 04 - nurse)	Fear to lose clinical and analytical skills
"I think it's important that you stand at the patient's bedside and look at the patient, that you don't just rely on some kind of monitoring." (interview 10 - physician)<br><br>*"Apart from that, I find that people play way too much with their phones, iPads and other stuff anyway. And if we even have such a thing at work, then I wouldn't have to get up at all anymore."* (interview 04 - nurse)	Fear of less direct contact with patients
"I honestly didn't think so much about this subject [future patient monitoring]*"* (interview 07 - respiratory therapist)	Lack of awareness or knowledge of intensive care

	staff about current technological developments
